# Self-control for IgE production

**DOI:** 10.18632/oncotarget.4806

**Published:** 2015-07-09

**Authors:** Brice Laffleur, Michel Cogné

**Affiliations:** UMR 7276 CNRS and Université de Limoges: Contrôle de la Réponse Immune B et Lymphoprolifération, Limoges, France

**Keywords:** Immunology and Microbiology Section, Immune response, Immunity

Immunoglobulin E (IgE) was the last discovered immunoglobulin class (in 1966). Having evolved from the same ancestral “IgY” gene duplicated in mammals 300 million years ago, IgG and IgE indeed followed very different careers with IgG now being the predominant serum Ig while IgE reaches levels 100,000-fold lower. In contrast, IgE became the most potent Ig, notably active against parasites, toxins and venoms, with ability to trigger vigorous or even violent immune reactions against antigens at nanomolar concentrations. Throughout evolution, it has clearly been an important issue for the immune system to maintain the production of such a powerful but dangerous weapon, which is responsible for severe anaphylaxis, under tight control [1].

Although class switch recombination (CSR) to Cε initiating IgE production is quite active *in vitro*, it is only weakly present *in vivo* due to various limitations (including inhibitory cytokines from the cell microenvironment, Cε gene-specific features with tightly regulated germline transcription and a poorly efficient acceptor switch region). For previously unknown reasons, IgE-switched lymphocytes additionally seem predisposed to a special fate, with boosted differentiation into short-lived plasma cells [2] and rapid *in vivo* apoptosis in germinal centers [3]. Previous studies of the elusive IgE^+^ B-cells *in vivo* thus relied on mice with helminth infections or transgenic models coupling IgE production to expression of a fluorescent marker. This notably demonstrated that Cε CSR can occur either directly from Cμ and then yield low affinity IgE antibodies, or from sequential CSR with a Cγ1/IgG1 intermediate, permitting somatic hypermutation and production of high affinity IgE. Whatever the pathway, IgE-switched B-cells further rapidly disappear for reasons that might relate to stimulation conditions (cytokines, T-cell help) or be B-cell intrinsic.

B-cell receptors (BCR), *i.e*. membrane anchored Ig (mIg), are major regulators of multiple aspects of cell physiology, such as cell survival, activation, proliferation, differentiation, and eventually activation-induced cell death [4]. There are clear indications that mIgM, mIgG and mIgA differentially support B cell survival and fate after activation, with mIgG and mIgA specially boosting plasma cell differentiation [5]. The IgE-class BCR has multiple peculiarities, including its transcription from a gene lacking a conventional polyadenylation site, the specific intracellular interactions of the mIgE tail, and its low expression level with alternatively spliced isoforms. We recently demonstrated that by itself, mIgE BCR directly restrains IgE production independently of the stimulation conditions and microenvironment [6]. This BCR-supported autonomous control of IgE^+^ lymphocytes was identified using different models for uncoupling mIgE expression and switch from the immune response and its cytokine/T-cell context, and was confirmed in normal primary cells and in hyper-Th2 LAT_Y136F_ mice. IgE-mediated B-cell elimination relies on a combination of original features, including spontaneous B-cell apoptosis and poor mobility across chemokine gradients [6]. Apoptosis notably involved the mitochondrial pathway and deregulated expression of several apoptosis controlling genes, including Phlda3, Card12 and Bim, and a unique sink effect of the mIgE tail which binds HAX-1 and disturbs the intra-cellular distribution of this anti-apoptotic factor. BrdU incorporation confirmed the short-lived fate of IgE^+^ cells, in agreement with their rapid collapse in transfer experiments. Transcriptomic analyses also indicated over-expression of Rgs13 (an inhibitor of GPCR signaling) and down-regulated expression of several genes controlling cell mobility (Plexin D1, S1pR1 and CXCL). Drastic changes were accordingly observed with regards to cell mobility against chemokine gradients (strongly decreased in transwell experiments) as well as cell morphology (mIgE^+^ cells become circular and lose pseudopods). Finally, IgE^+^ cells also showed spontaneously localization of the BCR in lipid-rafts, thus explaining its rapid internalization and turn-over, while intracellular phosphorylation was decreased. Interestingly in our model of tamoxifen-inducible / antigen-independent IgE class switching, mIgE+ cells were all short-lived but could yield plasma cells which were able to persist *in vivo* for a long time, most likely as long-lived plasma cells [6]. This work thus integrates previous studies which have explored the basis for the rarity of IgE^+^ B-cells *in vivo* but now identify the mIgE BCR itself as a major regulator of B-cell fate [2, 3, 7].

Long-term “IgE memory” is thus present in the plasma cell compartment allowing tiny amounts of soluble IgE to be secreted over the long term. In contrast, it is probably absent from the memory B-cell compartment, where it rather relies on adequately triggered *de novo* CSR of IgG^+^ memory B-cells (and of eventually some mIgM^+^ memory lymphocytes). These elements are clearly important for the design of future strategies to treat allergic patients. They also provide interesting new clues about the ability of lymphocytes to exercise a very active self-control and the molecular mechanisms involved.
